# MFAP2 is overexpressed in gastric cancer and promotes motility via the MFAP2/integrin α5β1/FAK/ERK pathway

**DOI:** 10.1038/s41389-020-0198-z

**Published:** 2020-02-13

**Authors:** Li-wen Yao, Lian-lian Wu, Li-hui Zhang, Wei Zhou, Lu Wu, Ke He, Jia-cai Ren, Yun-chao Deng, Dong-mei Yang, Jing Wang, Gang-gang Mu, Ming Xu, Jie Zhou, Guo-an Xiang, Qian-shan Ding, Yan-ning Yang, Hong-gang Yu

**Affiliations:** 1 Department of Gastroenterology, Renmin Hospital of Wuhan University, Wuhan, Hubei 430060 P.R. China; 2 Hubei Key laboratory of Digestive System, Renmin Hospital of Wuhan University, Wuhan, Hubei 430060 P.R. China; 3 Department of General Surgery, The Second People’s Hospital of Guangdong Province, Southern Medical University, Guangzhou, Guangdong 510317 P.R. China; 4 Department of Biochemistry, Zhongshan Medical College, Sun Yat-sen University, Guangzhou, P.R. China; 5 Department of Pathology, Renmin Hospital of Wuhan University, Wuhan, Hubei 430060 P.R. China; 6 Department of Ophthalmology, Renmin Hospital of Wuhan University, Wuhan, Hubei 430060 P.R. China

**Keywords:** Gastric cancer, Cancer microenvironment

## Abstract

Gastric cancer (GC) is one of the most common malignancies and its prognosis is extremely poor. This study identifies a novel oncogene, microfibrillar-associated protein 2 (MFAP2) in GC. With integrative reanalysis of transcriptomic data, we found MFAP2 as a GC prognosis-related gene. And the aberrant expression of MFAP2 was explored in GC samples. Subsequent experiments indicated that silencing and exogenous MFAP2 could affect motility of cancer cells. The inhibition of silencing MFAP2 could be rescued by another FAK activator, fibronectin. This process is probably through affecting the activation of focal adhesion process via modulating ITGB1 and ITGA5. MFAP2 regulated integrin expression through ERK1/2 activation. Silencing MFAP2 by shRNA inhibited tumorigenicity and metastasis in nude mice. We also revealed that MFAP2 is a novel target of microRNA-29, and miR-29/MFAP2/integrin α5β1/FAK/ERK1/2 could be an important oncogenic pathway in GC progression. In conclusion, our data identified MFAP2 as a novel oncogene in GC and revealed that miR-29/MFAP2/integrin α5β1/FAK/ERK1/2 could be an important oncogenic pathway in GC progression.

## Introduction

Gastric cancer (GC) is one of the most common and lethal malignant cancer throughout the world, particularly in Eastern Asian and South American countries^1^. Surgery is the optimal strategy of treating patients with GC; unfortunately, the application of surgical resection in patients with GC is limited, as most patients are diagnosed at an advanced stage of the disease^2^. What is more, many cases of GC are also not sensitive to chemotherapy and radiotherapy, making the situation more severe^2^. Recent years have witnessed the great progress of targeted cancer therapies; however, for GC patients, only trastuzumab, a monoclonal antibody against human epidermal growth factor receptor 2, and ramucirumab, a monoclonal antibody against vascular endothelial growth factor receptor 2, proved to have certain therapeutic effects and are widely applied in clinic^3,4^. Current treatment regimens for GC are still not adequate.

Researchers are trying to clarify the biological mechanisms underlying tumorigenesis and progression of GC, aiming to provide novel clues to fight against this fatal disease. With the rapid development of high-throughput detection techniques, gene expression data are accumulating rapidly in public repositories and a massive amount of differentially expressed genes (DEGs) between GC and normal tissue has been identified in several studies^5–9^. Many DEGs have been validated as oncogenes or tumor suppressors, which effect different malignant phenotypes of GC including proliferation, angiogenesis, metastasis, and chemoresistance via activating or inactivating multiple downstream signaling pathways^5–9^. But owing to different sample resources, experimental techniques, and bioinformatics algorithms, the results among these studies are greatly divergent, and there is still no widely accepted factor dominating the malignant transformation and progression of GC.

Integrative reanalysis of independent transcriptomic data may indicate common and remarkable changes during GC progression. In this study, by integrative analysis of datasets from either Gene Expression Omnibus (GEO) or The Cancer Genome Atlas (TCGA) databases, we successfully unveiled a set of DEGs that were invariably dysregulated in each cohort. Intriguingly, the functions of intersecting DEGs were found to significantly focus on the biological processes, such as extracellular space, extracellular matrix (ECM) organization, extracellular exosome, collagen catabolic process, and ECM–receptor interaction. ECM provides both the structure and signals that modulate biological behavior of cells, and recent studies have established the importance of the remodeling of ECM in cancer progression^10,11^. Our results implied that matrix remodeling was a hallmark of GC, which was probably underestimated in the past.

To further verify the crucial role of matrix remodeling in GC progression, we conducted survival analysis and obtained 14 genes associated with prognosis of GC patients, including SPARC, MFAP2, SERPINE1, LOX, PDGFRB, OLFML2B, VCAN, COLA18A1, SPON2, COL4A2, CHD11, NRP1, NREP, and COL4A5. Consistent with our expectations, most of them were important components of ECM or important modulators of matrix remodeling. This provided further evidence implying the crucial role of ECM in GC progression.

Among the 14 genes, we were particularly interested in MFAP2 (the microfibrillar-associated protein 2), which is also named microfibril-associated glycoprotein 1 (MAGP1). It is a 183-amino acid protein composed of two domains: a proline- and glutamine-enriched residues in amino terminal half and a 54-amino acid region in carboxy terminal half that targets itself to ECM^12,13^. Its extracellular form binds to fibrillin, collagen VI, tropoelastin, decorin, and biglycan^14^, and the intracellular form of MFAP2 upregulated the expression of downstream genes linked to cell adhesion, motility, and matrix remodeling^13^. Recently, the function of MFAP2 in metabolic disease has attracted a lot of attention. Previous studies demonstrated that, in adipose tissue, MFAP2 had high affinity for members of the transforming growth factor (TGF)-β superfamily, and in the absence of MFAP2, there was an increase in basal TGF-β activity^15,16^. However, its role in cancer biology is still obscure. In this study, we validated that MFAP2 was upregulated in GC tissue, and it was implicated in the malignant behavior of GC cells, such as proliferation, migration, and invasion. We also demonstrated that it activated focal adhesion kinase (FAK), paxillin, and extracellular signal-regulated kinase 1/2 (ERK1/2) through the MFAP2/integrin α5β1/FAK/ERK1/2 pathway. Furthermore, we explored the mechanisms of its expression dysregulation in GC. Loss of microRNA29 (miR-29) is known to be a mechanism of fibrosis and we found that MFAP2 was a target of miRNA-29 family, and its aberrant high expression was probably due to the absence or inhibition of miR-29 family.

In general, we reveal a set of GC-related genes that are potential diagnostic biomarkers and therapy targets. We also demonstrate that the novel oncogene MFAP2 endows cancer cells by activating integrin signaling. Finally, we provide evidence that miR-29 family members have potential to inhibit MFAP2 and at least partly reverse the aberrant matrix status of GC.

## Materials and methods

### Study strategy

**Fig. 1:**
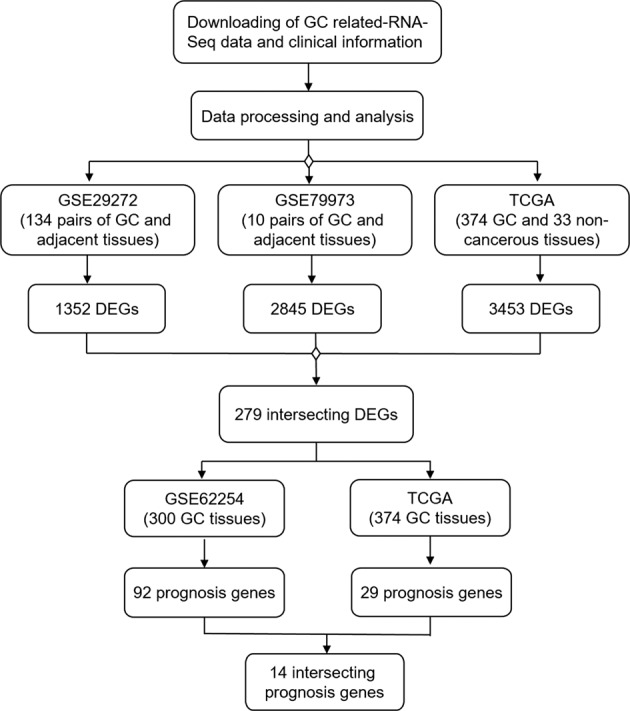
Workflow of data mining. Gastric cancer (GC)-related RNA sequence data were used to screen differentially expressed genes (DEGs) between GC and normal gastric tissues. After taking intersection from different cohorts, DEGs were further screened to identify prognosis-associated genes. Number of candidate genes remaining at each step is shown.

The workflow of data mining and the number of candidate genes remaining at each step are shown in Fig. 1.

### Patients and gene expression data

In this study, five cohorts of patients with GC (GSE29272, GSE79973, GSE62254 and GSE15459 and TCGA) were used for identifying and validating prognostic biomarkers. Description of these cohorts is presented in Supporting Information.

### Identification of DEGs

DEGs between matched GC and adjacent normal gastric tissues were identified using TwoClassDif^17,18^. Briefly, we first filtered DEGs with a fold-change (Tumor/Normal) of >1.5 or <0.67. Next, we confirmed the DEGs with the random variance model-modified *t* test to reduce statistical errors. Venn diagrams were drawn by online BioVenn website (http://www.biovenn.nl/index.php).

### Functional annotation

DAVID database and Gene Ontology (GO) functional and Kyoto Encyclopedia of Genes and Genomes (KEGG) pathway enrichment analysis were used to explore the potential biological function of intersecting DEGs from different cohorts^19^. *P* value < 0.01 and false discovery rate (FDR) <0.25 were set as the cutoff criteria.

### Identification of prognostic genes

Patients were classified as either high-expression (more than the median expression level of DEGs) or low-expression (less than the median expression level of DEGs) groups according to the expression of intersecting DEGs one by one. Univariate analyses of overall survival (OS) were performed with two-sided log-rank test to compare the differences between the two groups.

Kaplan–Meier plots were made using an online dataset (http://www.kmplot.com)^20^ with the data of GSE15459 and GSE62254. The analysis was performed using both disease-free survival (DFS) and OS information of patients. The patients were split by median.

### Lentivirus transfection

To knockdown the expression of MFAP2, we infected AGS and HGC-27 cells with the MFAP2–short hairpin RNA (shRNA) recombinant lentivirus (Genepharma, Suzhou, China). Detailed protocol of lentivirus transfection is presented in Supporting Information.

### Cell proliferation assay

MTT assay was performed using Thiazolyl Blue Tetrazolium Bromide (MTT, M2128, Sigma) following the manufacturer’s recommendations. The cell viability was detected using the multifunctional microplate reader at 490 nm with cells incubated for 2 h at 37 °C. The relative absorbance value was normalized and compared to the control group.

### In vitro migration and invasion assays

In the migration assay, cells were plated into the upper chamber of 8-mm-pore-size Transwell chambers (Corning, Corning, NY). Dulbecco's modified Eagle’s medium containing 10% fetal bovine serum was added into the lower chamber. Then the chambers were incubated at 37 °C for 48 h. Cells in the upper chamber were then removed, and the bottom surface of the membranes was counted using 0.1% crystal violet dye. In the invasion assay, matrigel (Clontech, Madison, WI) was used in the Transwell chambers (Corning). Cell migration and invasion were qualified by counting six random fields under a microscope.

### Immunofluorescence

AGS cells were grown to confluency on glass coverslips. Cells were fixed with 3.7% paraformaldehyde in phosphate-buffered saline for 20 min. Cells were permeabilized with 0.1% Triton X-100 for 5 min at 4 °C and then blocked with 5% bovine serum albumin in TBST for 1 h. Samples were incubated with primary antibodies overnight for 4 °C and then with appropriate secondary antibodies. Samples were mounted onto slides with mounting medium, and images were acquired using a fluorescence microscope. Images were processed using the Photoshop software (Adobe).

### Luciferase assay

Cells were plated into 24-well plates and cotransfected with 200 ng of psiCHECK-2 plasmids and 50 nmol/l of miR-29a (or NC microRNA) for 48 h. Luciferase activities were then measured using Dual-Luciferase Reporter Assay system (Promega, Madison, WI). Renilla luciferase activity was normalized to firefly luciferase activity.

### In vivo assays

Animal protocols were approved by the Institutional Animal Care and Use Committee of the Renmin Hospital of Wuhan University. Nude mice (4–5-week old) were raised in an specific pathogen-free environment at the experimental animal center of the Renmin Hospital of Wuhan University. Xenograft tumor growth models were established by subcutaneous injection of MFAP2 knockdown cells and NC cells (2 × 10^6^ cells) into the right dorsal flank. Tumor growth in the nude mice was observed for 28 days. Tumor volume (*V*, cm^3^) was evaluated based on tumor length (*L*) and width (*W*) with the following formula: *V* = 1/2 × *L* × *W*^2^. In order to test how MFAP2 affect tumor metastasis, we established metastatic tumor model by giving intravenous tail vein injections of 1 × 10^5^ MFAP2 knockdown cells to two groups of mice. After 7 weeks, the mice were sacrificed, and the tumor nodules formed on the lung and liver surfaces were counted. The tumors were embedded in paraffin for further study. All animal studies were conducted with the approval of the Renmin Hospital of Wuhan University and Use Committee.

### Statistical analysis

The correlation between gene expression and the clinicopathologic features was analyzed by Chi-square test using SPSS 20.0 (International Business Machines, Armonk, NY, USA). Three independent experiments were conducted in cellular studies, and results were analyzed using the two-tailed, unpaired Student’s *t* test. The mean standard deviation (SD) of three independent experiments was determined. Results were expressed as mean ± S.E.M. *P* < 0.05 was considered statistically significant.

## Results

### Identification of DEGs in GC

**Fig. 2:**
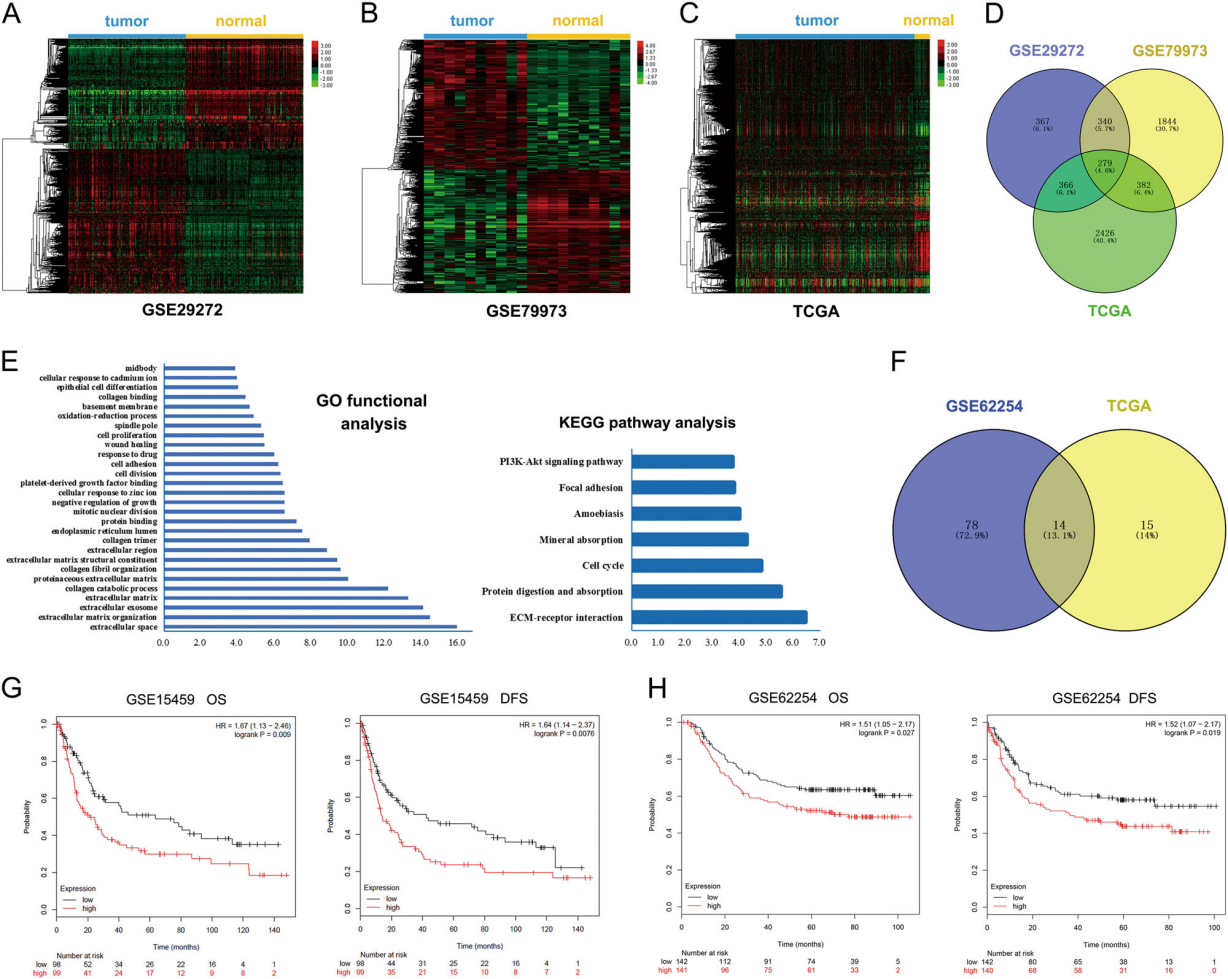
Identification of differentially expressed genes (DEGs) and prognosis-associated genes. **a**–**c** Using two-dimensional hierarchical clustering, 1352, 2845, and 3453 DEGs were identified from the expression profile datasets GSE29272 (*n* = 134), GSE79973 (*n* = 10), and TCGA (*n* = 374), respectively. **d** Taking the intersection of DEGs from the three datasets, 279 DEGs were extracted between GC and normal gastric tissues. **e** DEGs in intersection were mapped onto the DAVID database and subjected to Gene Ontology (GO) functional and Kyoto Encyclopedia of Genes and Genomes (KEGG) pathway enrichment analyses. GO function and KEGG pathway analysis show that DEGs in intersection are significantly associated with matrix remodeling process. **f** Using data with clinical information from GSE62254 (*n* = 300) and TCGA (*n* = 374), log-rank test was performed to explore the prognostic value of the intersecting DEGs. Ninety-two and 29 genes were closely related to patients’ overall survival in GSE62254 and TCGA, respectively. Taking the intersection of the two datasets, 14 prognostic biomarkers were obtained. **g** Among the 14 prognostic biomarkers, we are most interested in microfibrillar-associated protein 2 (MFAP2). Kaplan–Meier survival for overall survival (OS) and disease-free survival (DFS) of GC patients was performed. OS (*P* = 0.009) and DFS (*P* = 0.008) of GC patients in GSE15459 was significantly negatively associated with the expression of MFAP2. **h** OS (*P* = 0.027) and DFS (*P* = 0.019) of GC patients in GSE62254 was significantly negatively associated with the expression of MFAP2.

From the expression profile datasets GSE29272 (*n* = 134), GSE79973 (*n* = 10), and TCGA (*n* = 374), we extracted 1352, 2845, and 3453 DEGs, respectively. Two-dimensional hierarchical clustering showed a marked difference of expression modules of the DEGs (Fig. 2a–c). Taking the intersection of DEGs from the three datasets, we extracted 279 genes differently expressed in the GC tissues compared to normal tissues, including 171 upregulated and 108 downregulated genes (Fig. 2d, Table S1).

### The DEGs in intersection are significantly associated with matrix remodeling process

As shown in Fig. 2e, in GO functional analysis, biological processes of the 279 DEGs were found to focus on the extracellular space (*P* = 1.12 × 10^−16^), ECM organization (*P* = 3.20 × 10^−15^), extracellular exosome (*P* = 7.84 × 10^−15^), ECM (*P* = 5.12 × 10^−14^), etc. In KEGG pathway analysis, ECM–receptor interaction (*P* = 3.56 × 10^−7^), protein digestion and absorption (*P* = 3.03 × 10^−6^), cell cycle (*P* = 1.59 × 10^−5^), and focal adhesion (*P* = 1.63 × 10^−4^) were identified as significant pathways. Collectively, these results implied that the dysregulation of ECM-related proteins are common features in different cohorts.

### Identification of prognostic genes among the DEGs

Using data with clinical information from GSE62254 (*n* = 300) and TCGA (*n* = 374), we further explored the prognostic value of the 279 DEGs. As shown in Fig. 2f, 92 and 29 genes were closely related to patients’ OS in GSE62254 and TCGA, respectively. Taking the intersection of the two datasets, 14 prognostic biomarkers were obtained (Fig. 2f, Tables 1, 2). Most of the 14 genes were closely related to matrix remodeling, which further supported that matrix remodeling is a crucial character of GC progression. Among the 14 genes, most of them have been reported in GC such as the well-known oncogenes PDGFRB^21^, VCAN^22^, and COL18A1^23^, while there were also four genes, MFAP2, OLFML2B, NREP, and COL4A5, that have never been studied in GC. We are especially interested in MFAP2. Kaplan–Meier analysis in cohorts GSE15459 and GSE62254 showed that increased MFAP2 expression revealed poor OS and DFS in GC patients (Fig. 2g, h). Clinical pathology analysis showed that the expression level of MFAP2 was positively correlated with venous invasion and local invasion (Table S2).

**Table 1:** *P* value of the 14 prognosis genes in survival analysis.

Expression in GC		Gene symbol	*P* value of log-rank test	
			GSE62254	TCGA
Up		SPARC	0.004	0.011
		MFAP2	0.006	0.002
		SERPINE1	0.020	0.001
		LOX	0.044	0.002
		PDGFRB	0.004	0.000
		OLFML2B	0.004	0.033
		VCAN	0.039	0.000
Down		COL18A1	<0.001	0.001
		SPON2	0.023	0.012
		COL4A2	0.000	0.013
		CDH11	0.046	0.043
		NRP1	0.002	0.009
		NREP	<0.001	0.006
		COL4A5	0.000	0.029

**Table 2:** Functions of the 14 prognosis-related genes in gastric cancer.

Gene symbol	Description	Roles in matrix remodeling	Roles in cancer	Roles in GC	References
SPARC	A secreted protein, regulates extracellular matrix assembly and deposition, counter-adhesion, effects on extracellular protease activity, and modulation of cytokine signaling pathways	Regulates the assembly, organization, and turnover of the ECM by binding and modulating the deposition of multiple structural components and attenuating the activity of extracellular proteases	Functions as a tumor suppressor in most cancers but promotes metastasis in glioma and pancreatic cancers	Negatively correlated with clinicopathological factors of GC and inhibits VEGF-induced proliferation and metalloprotease-mediated metastasis of GC cells	^46–52^
MFAP2	Associates with fibrillin to create the functional form of the fiber; MFAP2-deficient mice suffered from multiple diseases, including hematostaxis, overweight, diabetes, osteohalsiteresis, and monopenia	Modifies microfibril function, interacts with the active form of TGF-β in ECM, and thus influences fibrosis/inflammation	Contributes to the progress of chronic obstructive pulmonary disease, which probably increases the risk of lung cancer; highly expressed in head and neck squamous cell carcinoma	N.A.	^13–16,53^
SERPINE1	A principal inhibitor of tissue plasminogen activator (tPA) and urokinase (uPA) and hence an inhibitor of fibrinolysis	Stimulates assembly of the fibronectin matrix and remodels the ECM through regulating plasmin	Plays a pro-tumorigenic role by promoting angiogenesis and inhibiting spontaneous apoptosis in cancer cells	Highly expressed in GC, associated with diffuse-type gastric cancer susceptibility	^54–58^
LOX	A secreted copper-dependent amine oxidase, initiates the crosslinking of collagens and elastin, and thus stabilizes fibrous deposits and contributes to tissue strength and integrity in the connective tissue	Cross-links fibers of collagen and elastin by oxidizing lysine residues into an aldehyde to form covalent unions among fibers. These unions insolubilize, stabilize, and harden the ECM	Overexpressed in most cancers and enhances cancer cell proliferation, invasion, metastasis, and angiogenesis. However, LOX has also been shown to have tumor-suppressor function in some studies	A tumor-suppressor gene in GC, associated with the epithelial–mesenchymal transition of GC cells	^59–64^
PDGFRB	A cell surface tyrosine kinase receptor for members of the platelet-derived growth factor family; essential for normal development of the cardiovascular system and aids in rearrangement of the actin cytoskeleton	Binds to integrins and TFG-β to modify ECM	Plays a pro-tumorigenic role by regulating mesenchymal cells of the tumor microenvironment of many common malignancies	Highly expressed in GC, and its overexpression is associated with unfavorable survival in GC patients; phosphorylation of PDGFR-β is correlated with depth of GC invasion	^21,65–68^^,^
OLFML2B	A member of the olfactomedin family, whose products are olfactomedin-domain-containing extracellular proteins capable of binding to proteoglycans, especially chondroitin sulfate-E and heparin	N.A.	N.A.	N.A.	^69,70^
VCAN	A large chondroitin sulfate proteoglycan and a major component of the extracellular matrix; involved in cell adhesion, proliferation, proliferation, migration, and angiogenesis and plays a central role in tissue morphogenesis and maintenance	Exhibits pro-fibrotic activity; forced expression of VCAN in cultured fibroblasts leads to the induction of the myofibroblast phenotype and the production of collagens	Promotes cancer cell motility, proliferation, and metastasis and is associated with poor outcome in many cancer types	Highly expressed in GC and promotes the progress of GC	^22,71–73^
COL18A1	A nonfibrillar collagen of basement membranes. The C-terminal fragment of the protein is termed endostatin and is a potent inhibitor of angiogenesis	Its degradation product, endostatin, interacts with laminin and regulates degradation and structure of basement membrane	Upregulated in most cancer; COL18A1 is degraded during the progression of tumor to release endostatin, an agent exerting an efficient inhibitory effect on tumor angiogenesis and growth	High expression of COL18A1 is associated with poor prognosis in patients with metastatic GC	^23,74–76^
SPON2	A secreted ECM proteins; essential for the initiation of immune responses and represents a unique a pattern-recognition molecule for microbial pathogens	Components of ECM	Unfavorable biomarker for some tumors, including gastric and prostate cancers	Highly expressed in GC and associates with clinicopathological features of GC	^77–80^
COL4A2	A subunit of type IV collagen, the major structural component of basement membranes; involved in hemorrhagic stroke and porencephaly	The C-terminal portion of the protein regulates various functions of myofibroblasts, ECM degradation, as well as stimulation of contractility in myofibroblasts	The C-terminal portion of the protein, known as canstatin, is an inhibitor of angiogenesis and tumor growth; COL4A2 is a diagnostic marker signature for esophageal and prostate cancer	COL4A2 is degraded during the progression of tumor to release canstatin, which induces GC cell apoptosis	^81–86^
CDH11	One of the type II classical cadherins, involved in cell–cell adhesion, potentially playing a role in development and maintenance of bone	Implicated in tissue morphogenesis and architecture, extracellular matrix-mediated tissue remodeling, cytoskeletal organization, and epithelial-to-mesenchymal transition	Promotes invasion and metastasis in prostate and breast cancers; in contrast, CDH11 is silenced by methylation and has a tumor-suppressor role in esophageal, colorectal, and gastric cancer	Silenced by methylation and has a tumor-suppressor role in GC	^87–89^
NRP1	A transmembrane protein essential for vascular and neural development and act as co-receptors for secreted signaling molecules of the class 3 semaphorin and vascular endothelial growth factor A families	Interacts with integrins and promotes integrin-mediated adhesion to ECM fibronectin	A mediator of tumor development and progression, extensively expressed in tumor vasculature; NRP1 overexpression is associated with tumor progression and poor clinical outcome	Promotes growth, invasion, and metastasis of GC; associated with clinicopathology of GC	^90–94^
NREP	Protein involved in cell–cell adhesion, potentially playing a role in neurite extension and nerve regeneration	N.A.	Promotes glioma motility and invasion through the reorganization of actin cytoskeleton at the cell periphery	N.A.	^95,96^
COL4A5	A subunit of type IV collagen, the major structural component of basement membranes; associated with X-linked Alport syndrome	Composes collagen and regulates fibrosis, organization, and morphology of tissues	Prevents cancer cell invasion, growth, and angiogenesis	N.A.	^97–100^

*N.A.* not applicable.

### MFAP2 is upregulated in GC tissues and cell lines

**Fig. 3:**
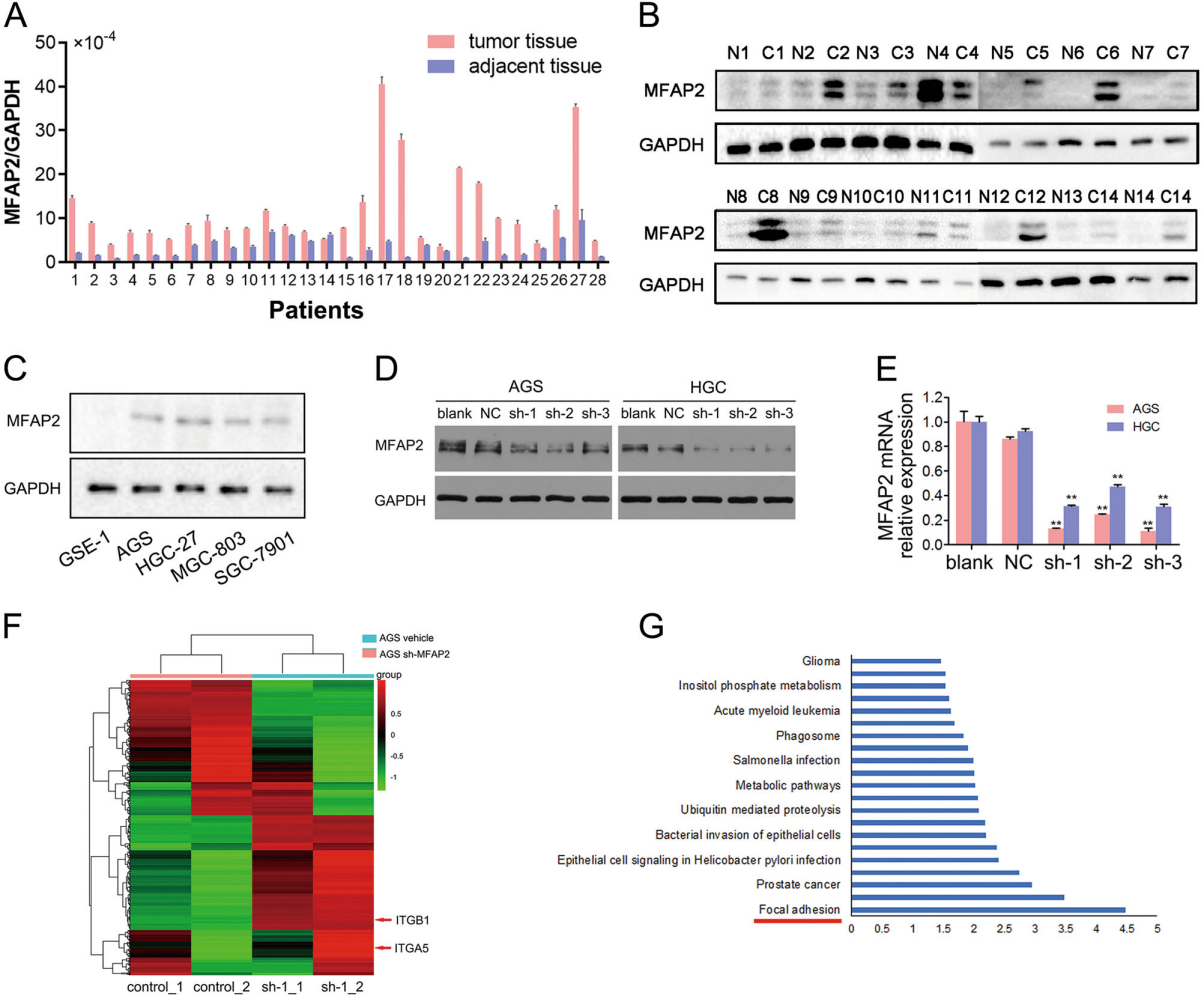
The RNA level of MFAP2 in clinical samples and sequencing analysis on MFAP2 knockdown cell lines. **a** Lysates from paired GC and adjacent normal tissues were analyzed by qPCR for the detection of MFAP2. GAPDH was used as a loading control. Each value presents the mean ± S.E.M. of three independent triplicate experiments. **b** Western blotting analysis of MFAP2 expression in 14 pairs of GC and adjacent tissues. **c** Western blotting analysis of MFAP2 expression in 4 GC cell lines, namely, HGC-27, SGC-7901, MGC-803, and AGS, and the normal gastric cell line GSE-1. GAPDH was used as a loading control. **d** The knockdown of MFAP2 in cells was affirmed by western blot. **e** The knockdown of MFAP2 in cells was affirmed by real-time RT-PCR. RNA sequencing using Illumina HumanHT-12 V4.0 expression beadchip was applied to assess the change of gene expression profile after MFAP2 knockdown in AGS cell line. **P < 0.01 vs. NC. **f** Cluster analysis of gene expression profile after MFAP2 knockdown. Downregulated (green) and upregulated genes (red) were identified. **g** Significantly changed pathways were identified based on Kyoto Encyclopedia of Genes and Genomes (KEGG) pathway database using the Gene Cloud of Biotechnology Information.

To validate the aberrant expression of MFAP2 in GC, we first observed the mRNA and protein levels of MFAP2 in 28 and 14 paired GC and adjacent tissues, respectively. As expected, most of the GC tissues exhibited significant upregulation of MFAP2 (Fig. 3a, b). Then we examined the expression level of MFAP2 in different GC cell lines and a gastric epithelial-derived cell line, GES-1. As shown in Fig. 3c, GC cell lines expressed more MFAP2 than GES-1. We also found that a previous study had verified that MFAP2 was upregulated in GC among 168 paired samples by immunohistochemistry^24^. These findings demonstrated that MFAP2 was upregulated in GC, implying the importance of them in GC pathogenesis.

### MFAP2 modulates integrin-stimulated FAK activation in GC

**Fig. 4:**
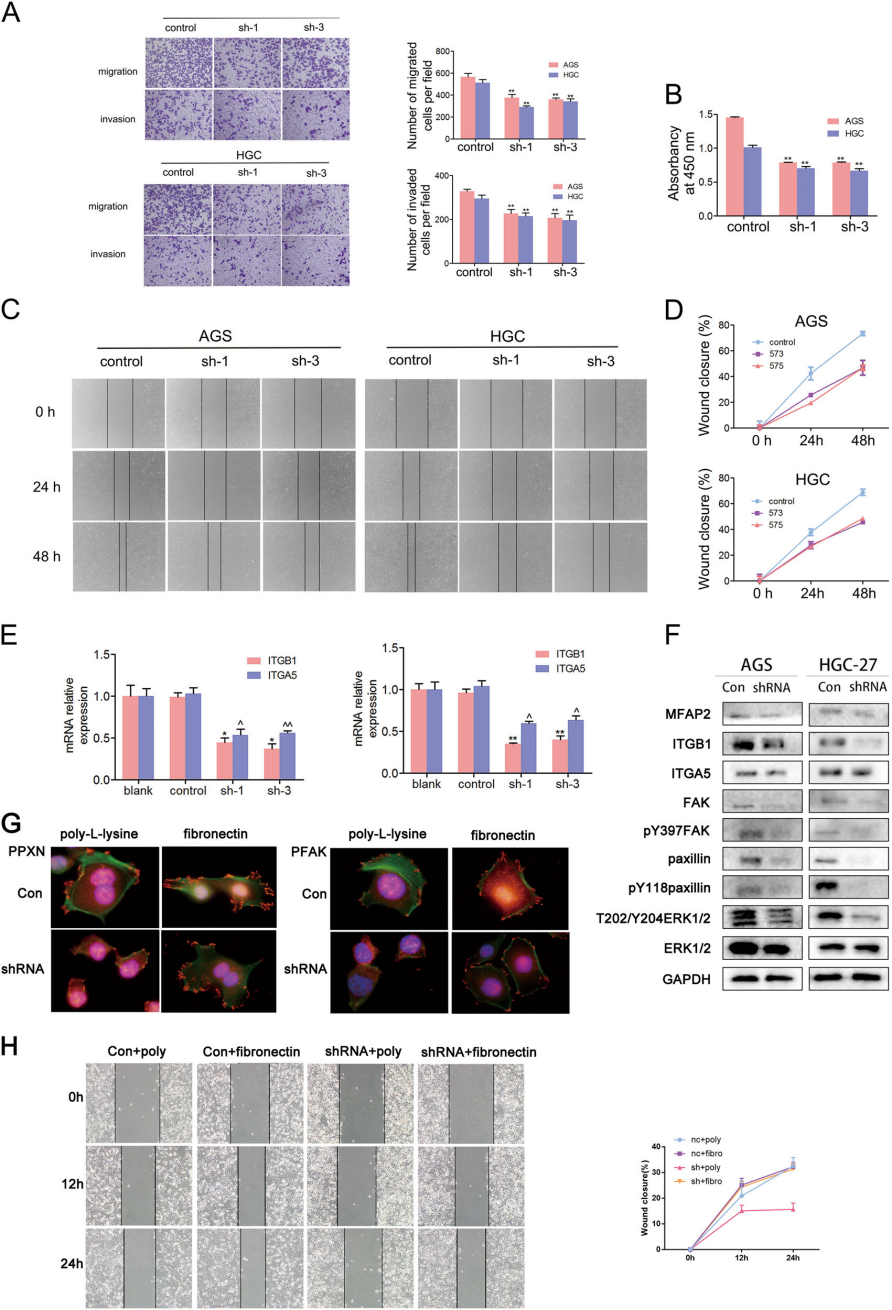
MFAP2 modulates proliferation, migration, and invasion of GC cells through integrin-stimulated focal adhesion kinase activation. **a** Cell migration was evaluated by cell wounding assay. **b** Wound width was quantified in eight fields per dish. **c** Cell migration and invasion were evaluated by migration and invasion assay. **d** The effect of MFAP2 knockdown on GC cell proliferation was determined by MTT assay. **e** Lysates from MFAP2 knockdown and scramble control cells were analyzed by qPCR to validate the downregulation of ITGB1 and ITGA5 after MFAP2 knockdown. GAPDH was used as a loading control. Each value presents the mean ± S.E.M. of three independent triplicate experiments. *P < 0.05 vs. blank, **P < 0.01 vs. blank, ^*P* < 0.05 vs. control, ^^*P* < 0.01 vs. control. **f** Western blotting analysis of MFAP2 knockdown and scramble control cells was performed to validate the downregulation of ITGB1 and ITGA5 and to explore the inhibition effect on FAK and paxillin after MFAP2 knockdown. **g** Fibronectin as a FAK activator was pre-added into Control and shMFAP2 cells. Immunostaining showed focal adhesion formation (×100, Green: F-actin, Red: pY118paxillin or pY397FAK, Blue: hoechst). **h** Wound healing assays showed that fibronectin significantly rescue AGS cells motility after MFAP2 knockdown (wound width was quantified in eight fields per dish).

In order to elucidate the molecular mechanisms by which MFAP2 influences GC pathogenesis, RNA sequencing was applied to assess the change of gene expression profile after MFAP2 knockdown in AGS cell line. First, we built a stably transfected MFAP2 knockdown cell line and validated it by western and quantitative PCR (qPCR; Fig. 3d, e). As shown by the sequencing, the control groups and MFAP2 knockdown groups were clearly distinguished by hierarchical cluster analysis (Fig. 3f). Five hundred and eighty-one DEGs were screened between sh-MFAP2 and control groups. Pathway analysis indicated that genes regulating focal adhesion were most significantly disrupted after MFAP2 knockdown (enrichment score = 6.82, *P* = 3.28 × 10^−5^, FDR = 0.005, Fig. 3g). Together with lamellipodia formation and cell polarization, adhesion to ECM via specific focal adhesion points has long been regarded as an essential step in cancer cell migration and invasion, which is mediated by integrin signal^25,26^. We noticed that both of ITGB1 and ITGA5 were downregulated after MFAP2 knockdown in RNA sequencing (Fig. 3e). This result was also validated by qPCR (Fig. 4e). Furthermore, western blotting was used to examine the expression of the integrin-stimulated FAK pathway in the MFAP2 Knockdown HGC-27 and AGS cells. As we expected, the expression of ITGB1, ITGA5, FAK, PXN, ERK1/2, PFAK (Tyr397), PPXN (Tyr118), and PERK1/2 (T202/Y204) were all prominently downregulated in the MFAP2 knockdown cells compared with the negative control (Fig. 4f). Based on the above study, we further explored FAK-associated phenotype on MFAP2 knockdown GC cell lines. As shown, HGC-27 and AGS cells displayed a lower cell proliferation rate than control cells after MFAP2 knockdown (Fig. 4b). What is more important, knockdown of MFAP2 suppressed the wound healing (Fig. 4c, d), migration, and invasion rates of AGS and HGC-27 cells (Fig. 4a). FAK is known as a crucial oncogene and promotes cell motility, survival, and proliferation, while in our current results, silencing of MFAP2 markedly inhibited FAK activation^27^. To explore whether the re-activation of FAK could rescue the effect of MFAP2 silencing, a widely used FAK activator fibronectin was applied^28^. As shown by immunostaining, the focal adhesion formation was significantly activated or re-activated by fibronectin (Fig. 4g). In the subsequent wound healing assays, AGS cells treated with fibronectin (10 μg/ml) showed stronger motility than the control group even after MFAP2 knockdown (Fig. 4h). Based on these results, we concluded that MFAP2 could possibly modulate integrin-stimulated focal adhesion formation and then work on motility and proliferation. Moreover, the effect of silencing MFAP2 could be rescued by activating FAK and paxillin.

### Altering MFAP2 expression has profound effects on tumor growth and metastasis in vivo

**Fig. 5:**
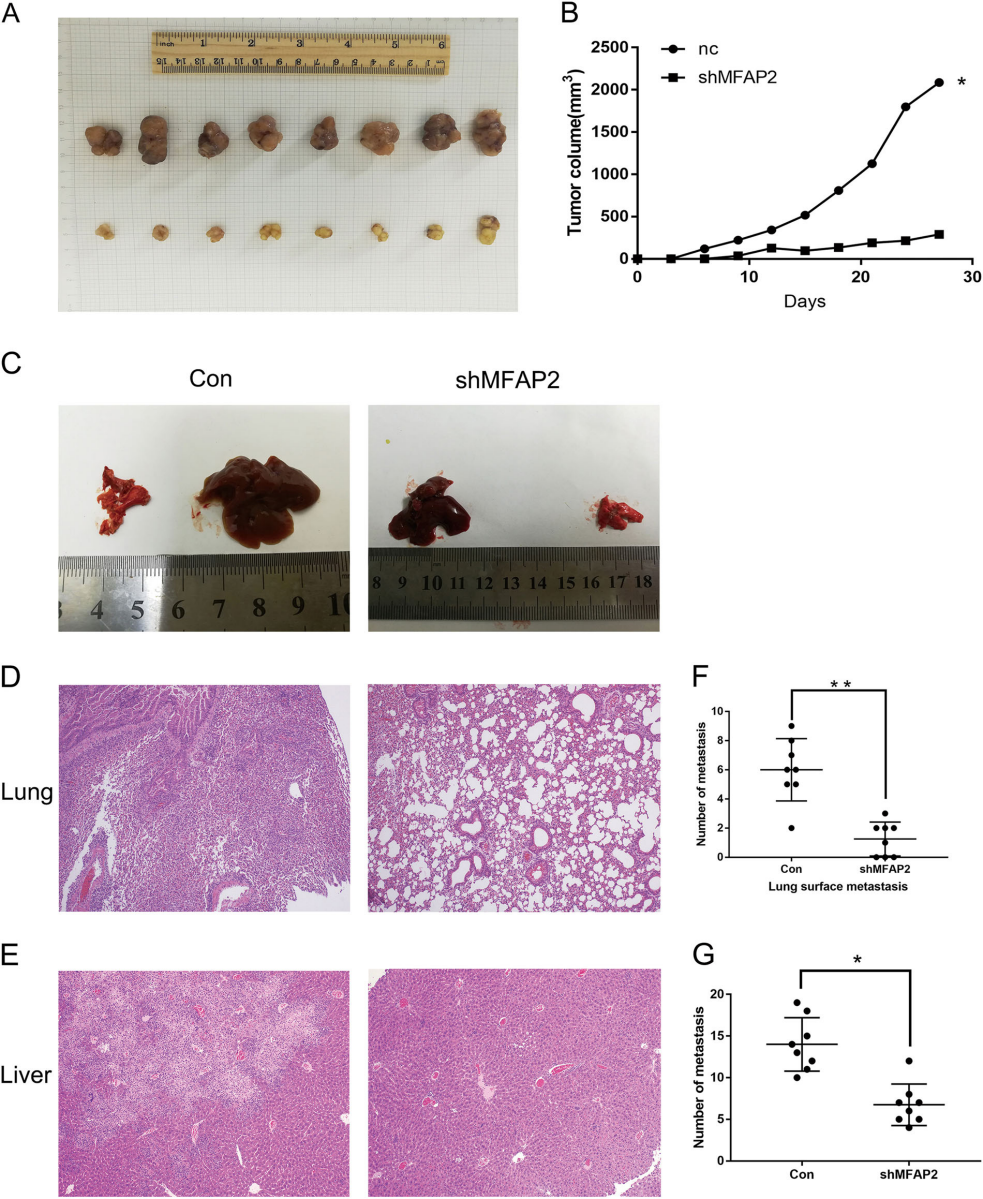
Silencing of MFAP2 by shRNA inhibited tumorigenicity and metastasis in nude mice. **a** Image of the xenograft tumors formed in nude mice injected with shRNA silencing of MFAP2 and scrambled shRNA control cells (NC). *P < 0.05. **b** Volume of xenograft tumors are summarized. **c** Representative images of metastatic tumor nodules in the lung and liver section of nude mice intravenously injected with MFAP2 knockdown and scrambled shRNA control cells (NC). **d**, **e** Representative images of H&E in the lung and liver section of nude mice (original magnification: ×40, calibration bar 125 μm). **f**, **g** Number of metastatic tumor nodules in the lung and liver are compared between nude mice injected with MFAP2 knockdown and scrambled shRNA control cells. **P < 0.01, *P < 0.05.

To better demonstrate influence of MFAP2 on proliferation and motility of GC cells, we performed nude mice xenograft assay and tail vein injection assay. For the xenograft assay, nude mice were subcutaneously injected with HGC-27 cells that had stably knocked down MFAP2 or empty vector as control. Tumor volumes were measured every 3 days after inoculation. Hematoxylin and eosin (H&E) staining was used to confirm that the nodules developed in mice were tumors (Table S3). Remarkably, we observed that the tumors formed by MFAP2 knockdown HGC-27 cells grew clearly slower than those formed by control cells (Fig. 5a, b). To evaluate the effects of MFAP2 on tumor metastasis in vivo, two groups of eight mice each were injected intravenously into the tail vein with MFAP2 knockdown or control cells, respectively. After 6 weeks, the mice were sacrificed, and the metastatic nodules in the lung and liver surfaces were counted. A significantly fewer number of metastatic nodules were induced at the surface of the lungs and livers of mice injected with the MFAP2 knocked down cells than in those with the control cells (Fig. 5f, g). H&E staining confirmed that the nodules on the surfaces of mice lungs and livers were metastatic tumors (Fig. 5d, e).

### Extracellular MFAP2 also modulates GC cells’ FAK activation

**Fig. 6:**
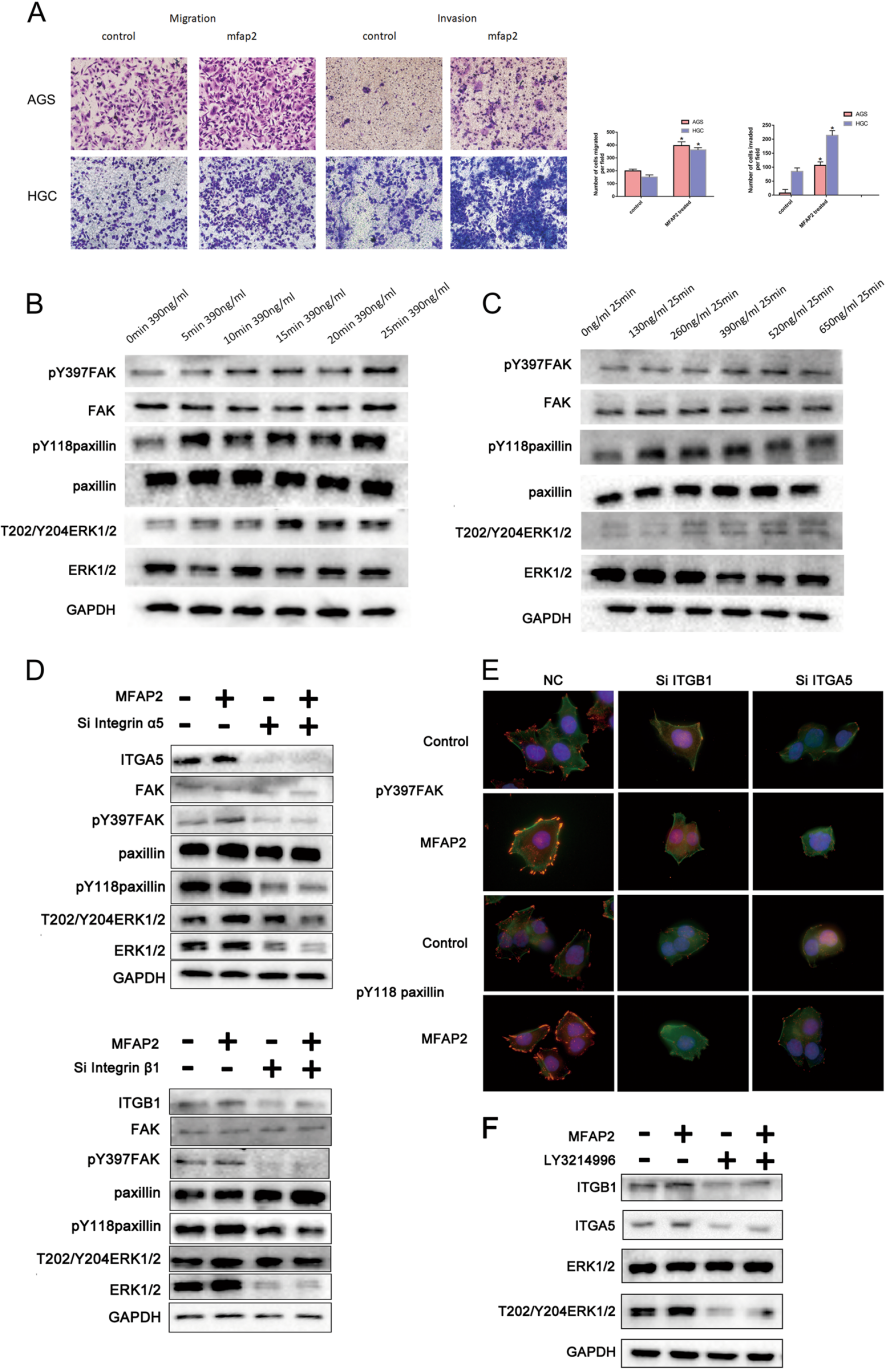
The extracellular MFAP2 activated focal adhesion kinase and regulated motility. MFAP2 recombinant protein was added into AGS and SGC-7901 cells. **a** Cell migration and invasion were evaluated by Transwell assay. **b** The effect of MFAP2 on FAK/paxillin/ERK tyrosine phosphorylation at different time gradients (390 ng/ml for 0, 5, 10, 15, 20, 25 min). *P < 0.05 vs. control. **c** The effect of MFAP2 on FAK/paxillin/ERK tyrosine phosphorylation at different concentration gradients (0, 130, 260, 390, 520, 650 ng/ml for 25 min). **d**, **e** Western blot and immunostaining showed the effect of MFAP2 on control or si ITGA5/ITGB1 AGS cell focal adhesion formation (×100, Green: F-actin, Red: pY118paxillin or pY397FAK, Blue: hoechst). **f** Western blot showed the effect of MFAP2 on DMSO or LY3214996-treated AGS.

According to previous researches and literatures, MFAP2 was an ECM protein whose function is to help structure elastic fibers^16^. So we explored whether the extracellular MFAP2 could influence focal adhesion formation. MFAP2 recombinant protein was added to the AGS and HGC-27 cell lines. Notably, treated with MFAP2 recombinant protein greatly enhanced migration and invasion rates of AGS and HGC-27 cells in Transwell assays (Fig. 6a). Then we added MFAP2 into AGS cell line at different concentrations and time gradients. As shown in picture, MFAP2 rapidly activated paxillin, FAK, and ERK1/2 in a time- and concentration-dependent manner (Fig. 6b, c). Immunostaining with pY118paxillin and F-actin showed that AGS cells rapidly made new focal adhesions after treating with MFAP2 recombinant protein (Fig. 6d, e). The expression of integrin α5β1 was reported to be correlated with ERK activity, and as an important downstream kinase of FAK, the activity of ERK1/2 was also closely related to MFAP2 knockdown or exogenous MFAP2 treatment^29,30^. Therefore, we interrogated whether the integrin expression was modulated in an ERK1/2 activation-dependent way. The AGS cell line was treated with an ERK1/2 inhibitor LY3214996 (200 nM) 2 h before exposure to MFAP2 recombinant protein. As shown, the expression of ITGA5 and ITGB1 significantly increased after MFAP2 treatment, while the expression remained unchanged when ERK1/2 inhibitor was pretreated (Fig. 6f). In conclusion, our results revealed that the extracellular MFAP2 was also strongly correlated with focal adhesion formation and mainly function through integrin α5β1 receptor. Moreover, MFAP2-modulated integrin expression was partially through ERK1/2 activation.

### Identification of MFAP2 as a novel target for miR-29

**Fig. 7:**
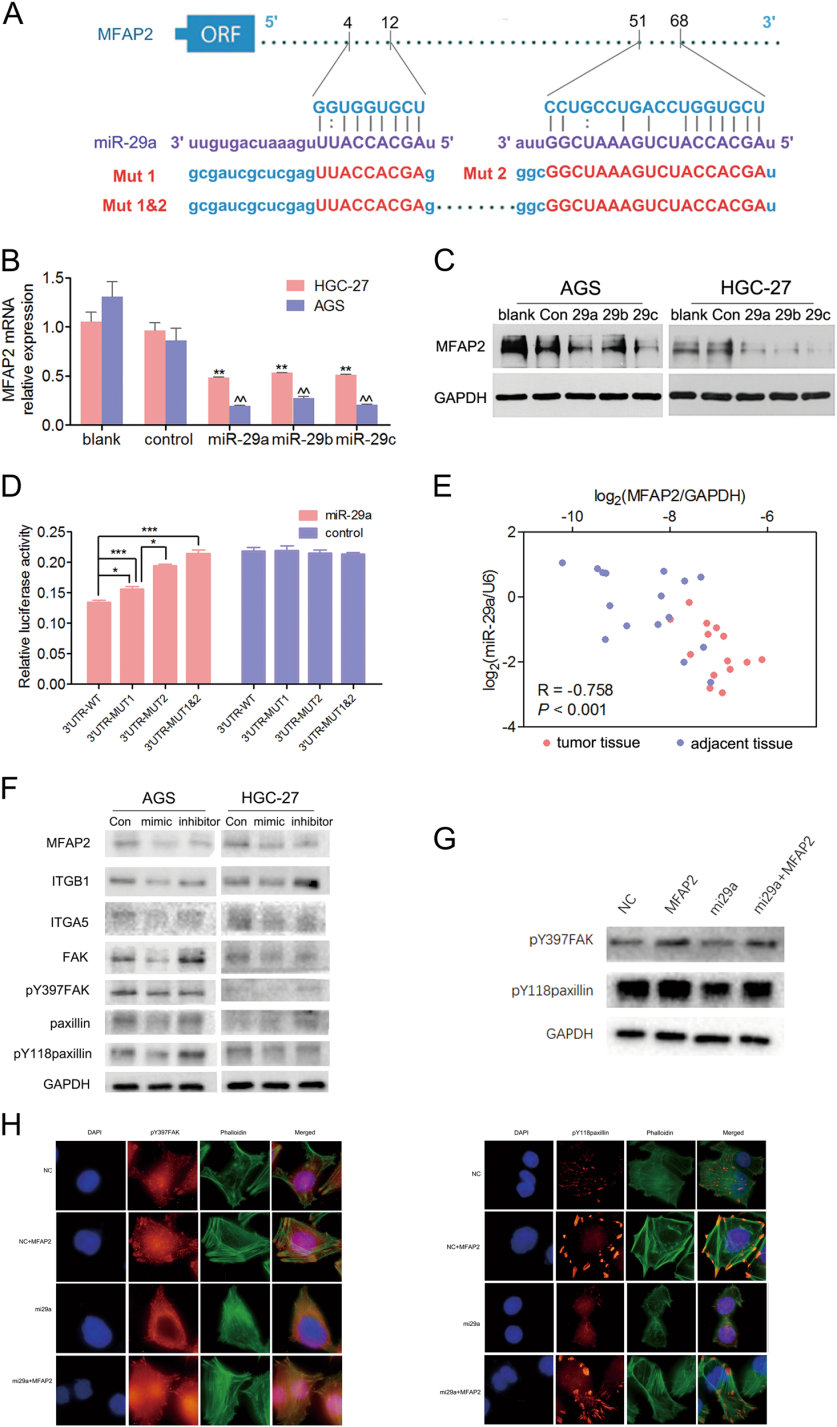
Identification of MFAP2 as a novel target for miR-29. **a** The target sites of miR-29a in 3’-UTR of MFAP2 are shown as a schematic representation. **b** Real-time RT-PCR showed the expression of MFAP2 mRNA in AGS and HGC-27 cells transfected with miR-29a mimics or inhibitors. **P < 0.01 vs. blank, ^^*P* < 0.01 vs. control. **c** Western blotting assays showed the expression of MFAP2 protein in AGS and HGC-27 cells transfected with miR-29a mimics or inhibitors. **d** Wild-type or mutant 3’-UTR constructs of MFAP2 were cloned into a psi-CHECK2 vector, respectively, and cotransfected with miR-29a mimics in HEK293 cells. Renilla luciferase activities were normalized to firefly luciferase activities. All assays were performed in triplicates and repeated at least three times. ***P < 0.001, **P < 0.01, *P < 0.05. **e** An inverse correlation was found between miR-29a expression and MFAP2 in GC samples (Spearman’s correlation, *P* < 0.001, *R* = −0.758). Each value presents the mean ± S.E.M. of three independent triplicate experiments. ***P < 0.001, **P < 0.01. **f** Western blotting analysis was performed to examine the expression change of ITGB1, ITGA5, FAK, and paxillin in AGS and HGC-27 cells transfected with miR-29a mimics or inhibitors. **g**, **h** Western blot and immunostaining showed that mi29a inhibited activation of FAK and paxillin while this inhibition could be rescued by MFAP2 (Green: F-actin, Red: pY118paxillin or pY397FAK, Blue: hoechst).

The mechanism of MFAP2 upregulation in GC tissues remained to be solved. We first investigated whether MFAP2 locus is amplified in human cancers using TCGA data on cBioPortal. However, the incidence of homozygous gain of MFAP2 locus was quite low (Fig. S1). We then explored whether the promoter region of MFAP2 was methylated low in human cancers using TCGA data available on MethHC. As shown, the methylation level of MFAP2 promoter was also not significantly decreased in cancer tissue (Fig. S2). Therefore, we focused on miRNA, another key factor contributing to gene expression regulation. Three databases, including TargetScan, miRanda, and miRWalk, were used to search potential microRNAs that are complementary to the 3’-untranslated region (UTR) of MFAP2. Only those miRNAs predicted by all of these methods were considered. One candidate identified was miR-29 family, whose family members miR-29a, miR-29b, and miR-29c all have two complementary sites to 3’-UTR of MFAP2 (located at 4–12 and 51–68, respectively, Fig. 7a, Fig. S3). miR-29 family is downregulated in GC^31,32^. After transfection with miR-29a, b and c, we observed that MFAP2 was downregulated on both mRNA and protein levels in AGS and HGC-27 cells (Fig. 7b, c). Among the miR-29 family, miR-29a transfection induced the most significant decrease of MFAP2 expression. Cells co-transfected with a miR-29a and wild-type MFAP2 3’-UTR presented a significant decrease in luciferase activity; however, in the mutant groups, much less changes in luciferase activity was observed (Fig. 7d). The luciferase reporter assay suggested that miR-29 family suppressed the transcription of the MFAP2 by targeting MFAP2. Further analysis of GC samples revealed the significant reciprocal association of expression levels between MFAP2 with miR-29a (Fig. 7e). FAK and paxillin are crucial integrin interactors and focal adhesion markers^25,33^. In AGS and HGC-27 cells, we observed that ITGB1, ITGA5, FAK, pY397FAK, paxillin, and pY118paxillin were downregulated after transfection with miR-29a mimics and upregulated after transfection with miR-29a inhibitors, which mimic the effects of MFAP2 knockdown or overexpression (Fig. 7f). To better visualize the correlation between MFAP2 and miR29a, we transfected AGS with miR-29a or scrambled control, and after 48 h, MFAP2 recombinant protein was added to activate the focal adhesion formation. As shown, treatment with MFAP2 could rescue the inhibition of miR29a on focal adhesion formation (Fig. 7g, h). Collectively, our results indicate that MFAP2 is a direct target of miR-29 family, and its dysregulation may have resulted from the loss of miR-29 family in GC.

## Discussion

Gene expression profile data were typically produced on a small scale in most studies, and the list of DEGs from different studies showed distressing inconsistency^5–8^. Even though some important “driver genes” in GC have been obtained from DEGs, excessive attention to single gene function may miss important changes of biological characters, which are often determined by a set of genes acting in concert^34^. Many hallmarks of cancer have been presented such as sustaining proliferation, resistance to cell death, enhancing angiogenesis, and activating metastasis^35^. In this study, via integrative reanalysis of several expression profile datasets, we identified a novel, generalizable hallmark of GC: matrix remodeling.

In this study, we obtained 14 genes associated with prognosis of GC patients. Most of them participate in matrix remodeling during cancer progression, including MFAP2. MFAP2 plays a vital role in the regulation of integrin signal pathway in cancer cell–ECM interaction. The intracellular form of MFAP2 can induce the transcription of integrin α4 in human osteosarcoma cell line SAOS-2^13^; in vascular development, MFAP2 defect will result in the reduction of integrin–matrix interaction^36^; our findings highlight the capability of MFAP2 to modify the phenotype of GC cells, partly by upregulating integrin α5β1. In this study, we concluded that MFAP2 might be an activator of integrin/FAK/ERK1/2 signaling in GC progression; however, it may also function importantly in GC carcinogenesis. In this work, there is only preliminary research on the role of MFAP2 in GC and many issues remain to be addressed. MFAP2 may exert oncogenic function by alternative mechanism. MFAP2 is associated with microfibrils in ECM and can also induce the expression of other matrix remodeling genes, such as VCAN^13^. Predictably, MFAP2 dysregulation will greatly change the status of ECM in cancer microenvironment and further modulate the phenotypes of cancer cells. Intriguingly, MFAP2 was also found to be an unfavorable indicator in multiple other cancers such as liver cancer, pancreatic cancer, renal cancer, and cervical cancer in our unpublished data. The mechanism of how MFAP2 exhibits functions in cancers remains to be studied in detail. In this study, we made a hypothesis that extracellular form of MFAP2 is produced by GC cells and promotes disease progression via autocrine secretion. Protein carriers between cancer cells and ECM such as exosome were potential mediators to transport MFAP2 to cancer microenvironment, and this need to be further explored and validated. Cancer-associated fibroblast is also an important regulator in matrix remodeling and whether it can secrete MFAP2 and participate in the deposition of MFAP2 are also worth studying. What is more, except for MFAP2, among these 14 genes, OLFML2B, NREP, and COL4A5 have not been investigated in GC previously, and they are also candidates for further functional studies.

MiRNA consists of short noncoding sequences that combine with target genes and inhibit gene expression by mRNA translational repression or degradation^37^. It has been demonstrated that miR-29 is downregulated in most cancers including GC^31,38–42^. Many downstream genes of miR-29 are matrix remodeling-related genes. For example, miR-29 mediates TGF-β1-induced ECM synthesis through activating Wnt/β-catenin pathway in human pulmonary fibroblasts^43^; in breast cancer tissues, miR-29 inhibits ECM network genes^44^; in pancreatic cancer, loss of miR-29 is correlated with a significant increase in ECM deposition^45^. In this study, we identify that MFAP2 is a direct target gene of miR-29, not only explaining the possible mechanisms of MFAP2 high expression in GC but also helping make the fact more explicit that miR-29 family is the crucial miRNA in matrix remodeling. A single miRNA can regulate multiple genes involved in a biological process. Some researchers think that, compared with conventional pharmacological approaches, the use of miRNA as a therapeutic agent may be more effective in some diseases^45^. Our results suggest that MFAP2 dysregulation partially resulted from loss of miR-29 family in GC, and miR-29-based therapy is promising in reversing aberrant ECM status of GC.

## Conclusion

In conclusion, our findings indicated that matrix remodeling is crucial in the development of GC. This study also provided potential biomarkers and therapy targets for GC and identified miR-29/MFAP2/integrin α5β1/FAK/ERK1/2 as an important oncogenic pathway in GC progression.

## Supplementary information

supplementary material legends

supporting information

Supplementary Figure 1. Cross-cancer summary of homozygous mutations and copy number variations of MFAP2 in all cancers available on cBioPortal.

Supplementary Figure 2. Cross-cancer summary of methylation level of MFAP2 promoter in all cancers available on MethHC.

Supplementary Figure 3. Target sites of miR-29b and miR-29c in 3’-UTR of MFAP2.

Supplementary Table 1. 279 differently expressed genes in gastric cancer tissues compared with normal tissues.

Supplementary Table 2. Correlation between MFAP2 expression and clinicopathological features in 300 patients with GC (GSE62254).

Supplementary Table 3. H&E of nude mice xenograft tumors.

## Data Availability

The datasets analyzed during the current study are available in the GEO repository with accession numbers GSE29272, GSE79973, GSE62254 and GSE15459 [http://www.ncbi.nlm.nih.gov/geo/] and the TCGA repository [https://cancergenome.nih.gov/]. The datasets generated during the current study are available in the GEO repository and assigned accession number GSE114796.
