# Supplementary material for: MFAP2 is overexpressed in gastric cancer and promotes motility via the MFAP2/integrin α5β1/FAK/ERK pathway
Source: Oncogenesis. 2020 Feb 13;9(2):17. doi: 10.1038/s41389-020-0198-z (PMC7018958; doi:10.1038/s41389-020-0198-z)
Supplement: Supplementary file 1 — supplementary material legends [file 41389_2020_198_MOESM1_ESM.docx]

**Supplementary legends:**

**Supplementary Table 1. 279 differently expressed genes in gastric cancer tissues compared with normal tissues.**

**Supplementary Table 2. Correlation between MFAP2 expression and clinicopathological features in 300 patients with GC (GSE62254).**

**Supplementary Table 3. H&E of nude mice xenograft tumors.**

**Supplementary Figure 1.** **Cross-cancer summary of homozygous mutations and copy number variations of MFAP2 in all cancers available on cBioPortal.** The incidence of homozygous gain of MFAP2 locus was quite low.

**Supplementary Figure 2. Cross-cancer summary of methylation level of MFAP2 promoter in all cancers available on MethHC.** The methylation level of MFAP2 promoter was not significantly decreased in cancer tissues.

**Supplementary Figure 3. Target sites of** **miR-29b and miR-29c in 3’-UTR of MFAP2.**
