# Supplementary material for: MFAP2 is overexpressed in gastric cancer and promotes motility via the MFAP2/integrin α5β1/FAK/ERK pathway
Source: Oncogenesis. 2020 Feb 13;9(2):17. doi: 10.1038/s41389-020-0198-z (PMC7018958; doi:10.1038/s41389-020-0198-z)
Supplement: Supplementary file 2 — supporting information [file 41389_2020_198_MOESM2_ESM.docx]

**Methods and materials**

**Description of gene expression data**

Four independent mRNA expression microarray datasets (GSE29272, GSE79973, GSE62254 and GSE15459) were downloaded from the GEO database ([http://www.ncbi.nlm.nih.gov/geo/) [5-9,17](http://www.ncbi.nlm.nih.gov/geo/)%20%5b5-9,17)]. Among these data sets, GSE29272 was based on an Affymetrix Human Genome U133A Array platform (GPL96), including 134 pairs of matched GC and adjacent normal gastric tissues. GSE79973 was based on an Affymetrix Human Genome U133 Plus 2.0 Array (GPL570), including 10 pairs of matched GC and adjacent normal gastric tissues. GSE62254 was based on GPL570, including 300 GC tumors with clinicopathological and prognostic information. TCGA database containing mRNA profile of 374 GC tumor and 33 non-cancers samples.

**Total RNA isolation and qRT-PCR**

Total RNA was isolated from paired GC/normal gastric tissues and cells using TRIzol reagent (Invitrogen, Carlsbad, CA, USA) according to the manufacturer’s instructions. 1 μg of RNA was reverse transcribed into 20 μl of cDNA using a reverse transcription kit (Promega, Madison, WI, USA). PCR amplification was performed with the CFX96 Real Time System (Bio-Rad, Hercules, CA) using SYBR Premix Ex Taq^TM^ (Takara, Japan). relative gene expression levels were calculated using the 2−ΔΔCt method. GAPDH was used as the internal control. Primers used in the present study are as following: MFAP2, forward 5′-CCAACCCCAGAACCAGGAAA-3′ and reverse 5′-CACGGCGGAGGCTGTAGAAG-3′; miR-29a, forward 5′-TAGCACCATCTGAAATCGGTTA -3′; miR-29b, forward 5′-TAGCACCATTTGAAATCAGTGTT -3′; miR-29c, forward 5′-TAGCACCATTTGAAATCGGTT-3′; miR-29a/b/c reverse: Uni-miR qPCR primer; ITGB1, forward 5′- GTTCAGTTTGCTGTGTGTTTGC-3′ and reverse 5′- ATCCTCTGGCTTGAGCTTCTCT-3′; ITGA5, forward 5′- GCTCTAAGCCTTCCCAGGTTCT-3′ and reverse 5′- GAGGCACTAGCGGACACGAT-3′; and GAPHD, forward 5′-CCCATCACCATCTTCCAGGAG-3′ and reverse 5′-CTTCTCCATGGTGGTGAAGACG-3′.

**Cell culture**

GC cell lines AGS, HGC-27, MGC-803, SGC-7901 were obtained from Procell Life Science & Technology Co.,Ltd (Wuhan, China). GC cell lines were maintained in Dulbecco’s modified Eagle medium (Gibco, 11320033) with 10% fetal bovine serum (Gibco, 10099141C). Procell has authenticated cell lines before our using according to STR fingerprinting and provide corresponding certificates (Wuhan, China).

**Western blotting**

The total proteins of paired GC/normal gastric tissues and cells were extracted, and protein concentration was determined using Bradford method. 20 μg protein extracts were electrophoresed in 10% SDS-PAGE, and transferred onto nitrocellulose membrane (Millipore, Billerica, MA). After blocking with 5% nonfat milk, the membranes were incubated with the primary antibodies against MFAP2, ITGB1 (Cell Signaling Technology, Beverly, US, #34971), ITGA5 (Cell Signaling Technology, Beverly, US, #98204), FAK (Cell Signaling Technology, Beverly, US, #3285), Py397FAK (Abcam, Cambridge, MA, #ab81298), paxillin (Invitrogen, Carlsbad, CA, USA, #ab32084), pY118 paxillin (Invitrogen, Carlsbad, CA, USA, #44-722G), ERK1/2 (Santa Cruz Biotechnology, Dallas, TX, #sc-514302), T202/Y204 ERK1/2 (Santa Cruz Biotechnology, Dallas, TX, #sc-81492), and GAPDH (Santa Cruz Biotechnology, Dallas, TX, #sc-47724) overnight at 4 °C. Subsequently, membranes were incubated with horseradish peroxidase (HRP)-labeled secondary antibody (Cell Signaling Technology, Beverly, US) for 1 h. Then the signals were detected using an enhanced chemiluminescence kit (Thermo, San Jose, CA).

**Lentivirus transfection**

Cells were plated in six-well plates at a confluence of 20-30%. After incubation of 12 h, we infected cells with MFAP2 sh-RNA or scrambled control-shRNA-expressing lentiviruses (Genepharma, Suzhou, China) using a supernatant fluid containing an appropriate viral titer. The viral supernatant was replaced with fresh media after incubating at 37°C for 12 h. The infected cells were selected using 10 μg/ml puromycin in DMEM (Sigma-Aldrich, St Louis, MO) for 4 weeks. Successful infection was examined *via* expression of green fluorescent protein observed by an inverted fluorescence microscope (Leica DMI4000 B; Leica Microsystems GmbH, Wetzlar, Germany).

**Analysis of microarray data**

The gene expression signals were normalized using log_2_ transformation. Hierarchical clustering was performed with average linkage. DEGs were filtered by the RVM (Random variance model) f-test, and clustered heatmap was visualized using Treeview. We selected the DEGs according to threshold set at *p* < 0.01 and FDR < 0.05.

Significantly changed pathways were identified based on Kyoto Encyclopedia of Genes and Genomes (KEGG) pathway database using the Gene Cloud of Biotechnology Information (GCBI Platform, Shanghai, China). Fisher's two-side exact test and Chi-square test were used, and signaling pathways were selected at threshold of *P* < 0.01 and FDR < 0.05.
