# Supplementary figures and images for: MFAP2 is overexpressed in gastric cancer and promotes motility via the MFAP2/integrin α5β1/FAK/ERK pathway
Source: Oncogenesis. 2020 Feb 13;9(2):17. doi: 10.1038/s41389-020-0198-z (PMC7018958; doi:10.1038/s41389-020-0198-z)

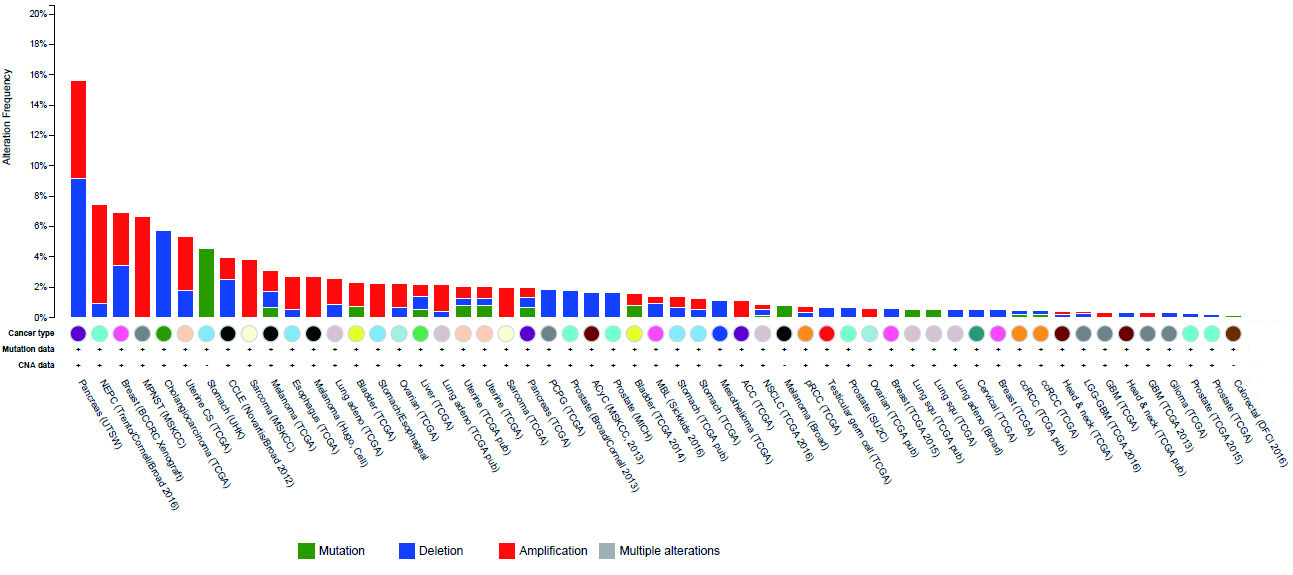

Supplement: Supplementary file 3 — Supplementary Figure 1. Cross-cancer summary of homozygous mutations and copy number variations of MFAP2 in all cancers available on cBioPortal. [file 41389_2020_198_MOESM3_ESM.jpg]

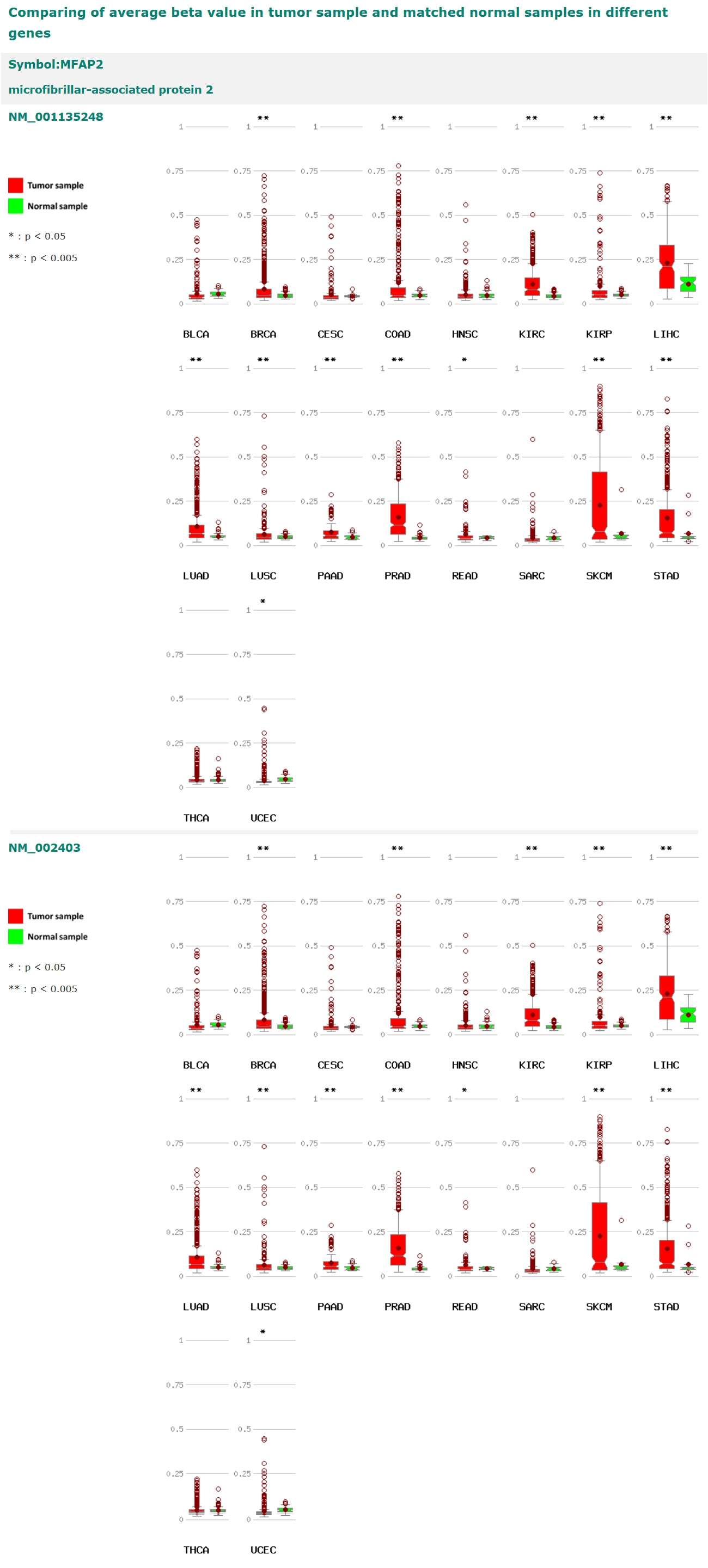

Supplement: Supplementary file 4 — Supplementary Figure 2. Cross-cancer summary of methylation level of MFAP2 promoter in all cancers available on MethHC. [file 41389_2020_198_MOESM4_ESM.jpg]

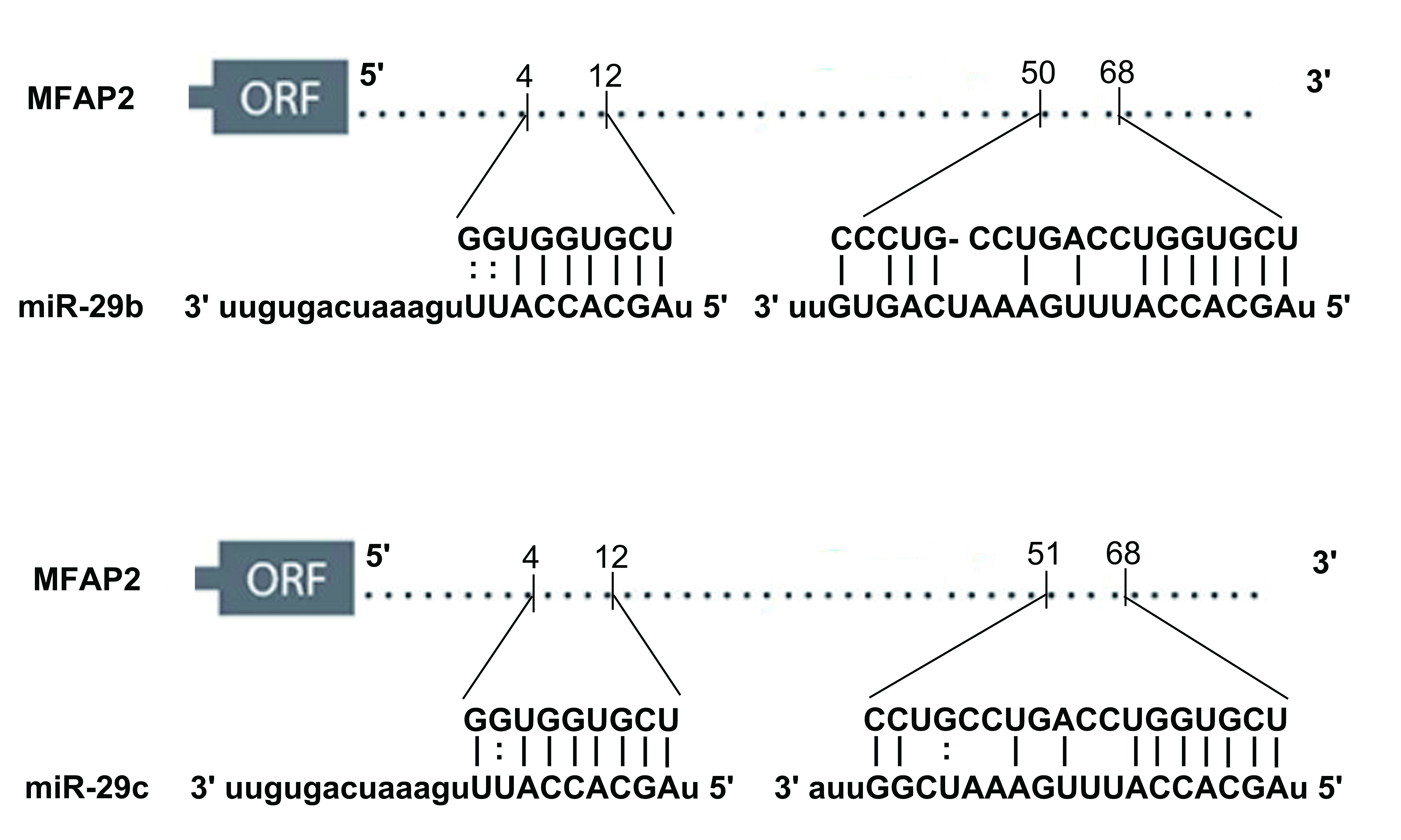

Supplement: Supplementary file 5 — Supplementary Figure 3. Target sites of miR-29b and miR-29c in 3’-UTR of MFAP2. [file 41389_2020_198_MOESM5_ESM.jpg]

**Table S3. H&E of nude mice xenograft tumors.**

Xenograft tumors: 40×，125um.


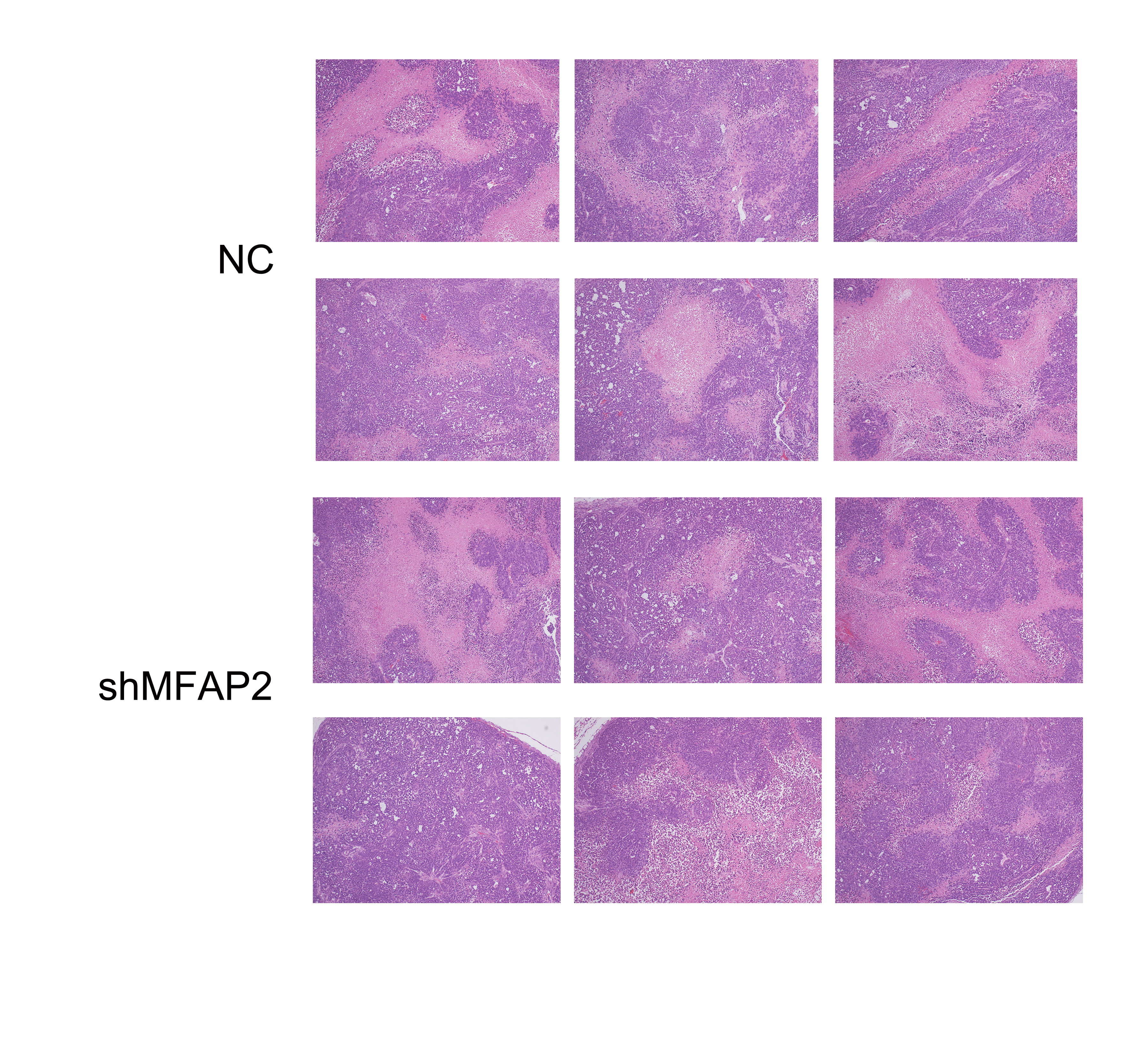

Supplement: Supplementary file 8 — Supplementary Table 3. H&E of nude mice xenograft tumors. [file 41389_2020_198_MOESM8_ESM.docx]
