# Supplementary material for: MFAP2 is overexpressed in gastric cancer and promotes motility via the MFAP2/integrin α5β1/FAK/ERK pathway
Source: Oncogenesis. 2020 Feb 13;9(2):17. doi: 10.1038/s41389-020-0198-z (PMC7018958; doi:10.1038/s41389-020-0198-z)
Supplement: Supplementary file 6 — Supplementary Table 1. 279 differently expressed genes in gastric cancer tissues compared with normal tissues. [file 41389_2020_198_MOESM6_ESM.docx]

**Table S1.** 279 differently expressed genes (DEGs) in gastric cancer tissues compared with normal tissues.

| DEGs | Gene Symbols |
| --- | --- |
| Up-regulated | TEAD4, ECT2, E2F3, KPNA2, RFC3, COL1A1, SERPINH1, RUVBL1, MCM4, BGN, HMGB3, TIMP1, MYO1B, CSE1L, MEST, BOP1, NOP56, SEPT11, BUB1, COL4A1, ARPC1B, PRC1, NUP62, ANP32E, COL5A2, SNX10, CEP55, PLAU, TPX2, SULF1, CKS1B, CDC25B, SKP2, ENO1, COL3A1, KIF2C, TOP2A, DTL, PKM, KIF4A, TRIP13, AGPS, SPARC, AGO2, CLDN7, MAD2L1, COL1A2, CENPF, DLGAP5, NEK2, PTPN12, MKI67, MELK, LY6E, UBE2C, GINS1, CTSB, APOE, CDK1, BUB1B, MAP4K4, CENPA, PMEPA1, NDC80, THY1, KIF20A, MFAP2, FCGR3A, MXRA5, CCNB2, TMEM185B, SERPINE1, LOX, S100A10, TACC3, CDC6, CCNB1, PDGFRB, FXYD5, SPP1, OLFML2B, MMP11, VCAN, FERMT1, OSMR, THBS2, JUP, FAR2, STIL, STK3, ATP1B3, GGH, IL32, CDC20, CXCL1, CTSZ, FCER1G, CXCL9, BIRC5, COL18A1, APOC1, FKBP10, PTPRG, COL5A1, FCGR2A, COL6A3, CDKN3, FSCN1, VASH1, GLS, SPON2, CXCL8, KDELC1, FGFR4, TRIO, COL4A2, ASPN, IGFBP7, CDH11, SOD2, FN1, CTSL, LUM, SFRP4, TM4SF1, NRP1, FZD2, CHI3L1, SQLE, KIF15, CCNE1, ASPM, RARRES1, MSR1, INHBA, TPBG, DACT1, MMP1, NREP, HOXB7, PLEKHS1, MMP7, TMEM158, LIPG, CST1, HIST1H2BJ, LGR5, WNT5A, TMEM45A, CCL18, LOXL1, NID2, PLXNC1, DIO2, TGM2, PLA2G7, PRRX1, QPCT, LEF1, CPVL, EPHB2, THBS1, GREM1, NNMT, CAMK2N1, TNFRSF25, TNFAIP6, SERPINB5, CDH3, SLAMF8, TNFRSF11B |
| Down-regulated | DPT, GPX3, SLC25A4, CBR1, ALDH6A1, ELL2, HPGD, ALDH3A1, MT1X, HBB, METTL7A, KLF4, HADH, SNRPN, CKB, SLC7A8, MT1M, ACACB, GKN1, CYB5R1, SIDT2, MAOA, DHRS7, CYP2C18, AKR1C1, GPD1L, HRASLS2, ADTRP, ACO2, PER3, KIT, SULT1B1, UBL3, POU2AF1, NR3C2, PDGFD, SEPP1, CYB5A, NMRK1, ALDOB, ADH1B, NR0B2, AKR7A3, PSCA, FMO5, PBXIP1, CD79A, GSTA4, PXMP2, SELENBP1, ALDH2, TOX, KLF2, RAB27A, GLUL, MT1F, AADAC, COL4A5, LIPF, C1ORF116, MT2A, MT1E, PLLP, FMO4, EPN3, AKR1B10, VILL, FAM46C, GSTA1, EPB41L4B, GCNT1, CYP4F12, PGC, KRT20, RNASE1, IGHA2, IGJ, RIPK4, PDCD4, CYFIP2, OXCT1, ERO1LB, SGK1, RORC, TFF1, PLCXD1, LTF, PBLD, ID1, MT1G, BCAS1, SULT1C2, FCGBP, PTGER4, C4orf19, FOS, IL1R2, AZGP1, IGFBP2, PLAC8, MT1H, APLP1, KIAA1324, SCNN1A, MUC5AC, CA2, CXCL14, MSMB |

DEGs: differently expressed genes.
