# Supplementary material for: MFAP2 is overexpressed in gastric cancer and promotes motility via the MFAP2/integrin α5β1/FAK/ERK pathway
Source: Oncogenesis. 2020 Feb 13;9(2):17. doi: 10.1038/s41389-020-0198-z (PMC7018958; doi:10.1038/s41389-020-0198-z)
Supplement: Supplementary file 7 — Supplementary Table 2. Correlation between MFAP2 expression and clinicopathological features in 300 patients with GC (GSE62254). [file 41389_2020_198_MOESM7_ESM.doc]

Supplementary Table 2. Correlation between the MFAP2 protein expression and differentiation status in 45 gastric cancer samples.

|  | No. of Patient | | Differentiation status  Well Moderate Poor | | | | Fisher’s exact test  *P* value | |
| --- | --- | --- | --- | --- | --- | --- | --- | --- |
| IHC result | |  | |  |  |  | |  |
| Strongly positive (+++) | | 16 | | 2 | 2 | 12 | | 0.000432 |
| Moderately positive (++)  Weakly positive (+) | | 17  12  45 | | 4  3  9 | 12  7  21 | 1  2  15 | |  |
